# T cell extravasation: Demonstration of synergy between activation of CXCR3 and the T cell receptor

**DOI:** 10.1016/j.molimm.2009.08.021

**Published:** 2009-12

**Authors:** Peter Newton, Graeme O’Boyle, Yvonne Jenkins, Simi Ali, John A. Kirby

**Affiliations:** Applied Immunobiology and Transplantation Research Group, Institute of Cellular Medicine, The Medical School, University of Newcastle, Newcastle upon Tyne NE2 4HH, UK

**Keywords:** CXCR3, T cell receptor, Endothelium

## Abstract

Endothelial cells present chemokines to T cells and can also stimulate the T cell antigen receptor by presentation of peptide–MHC antigen complexes. This study was designed to investigate the potential synergy between stimulation of the chemokine receptor CXCR3 and the human T cell receptor complex. Transendothelial T cell migration towards CXCL10 was modified by crosslinking CD3 immediately before addition to the endothelium. When resting endothelium was used, T cells which had been activated by crosslinking CD3 for only 1 min showed a significant reduction (*p* < 0.0001) in migration when compared with untreated T cells. By contrast, endothelial cells which had been activated by stimulation with interferon-γ and tumour necrosis factor-α supported a specific increase in the migration of activated T cells; this was most apparent after CD3 had been activated for 90 min (*p* < 0.0001). The molecular basis for synergy between CXCR3 and the T cell receptor complex was investigated by measurement of fluorescence resonance energy transfer. This showed that CXCL10 induced a close (<10 nm) spatial association between CXCR3 and the CD3ɛ subunit on the cell-surface. These data demonstrate that stimulation of both CXCR3 and the T cell receptor has the potential to enhance specifically both the proliferation and extravasation of specific T cells during episodes of local inflammation.

## 1 Introduction

Transendothelial migration of leucocytes from the blood into sites of tissue inflammation is fundamental to the effective operation of the immune system. A multistep model for such migration is now generally accepted (Adams and Shaw, 1994). Leukocytes initially utilise relatively low affinity, selectin-mediated adhesion to support shear-stress induced rolling contact with the luminal surface of blood vessels. Subsequent encounter with inflammatory chemokines induces integrin activation resulting in leukocyte arrest. This is followed by cell flattening and the development of processes which identify intercellular junctions that can support diapedesis (Ley et al., 2007).

Integrin activation is mediated by the stimulation of G-protein coupled receptors on the surface of leucocytes by heparan sulphate-immobilised chemokines on the apical surface of endothelial cells (Hardy et al., 2004). There is differential expression of chemokine receptors by functional subsets of leukocyte (Campbell et al., 2003; Sato et al., 2007), and differential chemokine expression within specific functional compartments and inflammatory sites. This permits the selective recruitment of appropriate immune cells to specific locations.

Although appropriate for most leukocytes, the multistep model takes no account of the antigen specificity of T cells. T cells recognise cognate antigen via the T cell receptor (TCR) when it is presented in the context of cell-surface MHC molecules by either specialised or non-specialised antigen presenting cells (Huppa and Davis, 2003). The ability of antigen recognition to influence T cell motility and integrin-mediated adhesion has long been recognised (Dustin and Springer, 1989; Epler et al., 2000), but the potential for it to influence transendothelial migration remains a subject for investigation.

Vascular endothelial cells express high levels of MHC class I and variable levels of MHC class II; both of these can be increased by inflammatory mediators such as IFNγ (Shiao et al., 2007). These cells can also take up protein antigens and process peptides for presentation to specific T cell receptors in both classes of MHC antigen (Savage et al., 1995). Although human endothelial cells do not express costimulatory B7 (CD80 or CD86) molecules, they are capable of inducing specific proliferation of CD4+ and CD8+ memory T cells (Al-Lamki et al., 2008).

A range of studies *in vitro* have focused on the effect of cognate antigen recognition on transendothelial T cell migration. Depending on the precise system used, the results have been variable. Initial studies suggested increased migration across endothelium which had been pre-treated with IFNγ (Marelli-Berg et al., 1999; Tay et al., 2003), whilst antigen-stimulated T cells were retained by resting endothelium (Tay et al., 2003). These studies did not define a role for chemokines in this system, although stimulation of endothelial cells with IFNγ is known to increase both the production (Sana et al., 2005) and heparan sulphate-mediated capture (Carter et al., 2003) of chemokines for apical presentation (Hardy et al., 2004) to immune cells. In particular, the IFNγ inducible chemokine CXCL10 is produced at a high level by activated endothelial cells (Luster and Ravetch, 1987).

More recent studies of rapid T cell migration across activated endothelial cells in the presence of shear-stresses, TCR stimulation and chemokines have revealed further complexity. Stimulation of the TCR was shown to reduce CXCL10-driven T cell migration across TNFα activated human umbilical vein endothelial cells (Manes et al., 2007). However, TNFα stimulated dermal microvascular endothelial cells supported enhanced T cell migration in the presence of TCR stimulation and the chemokine CX3CL1 (fractalkine), which is expressed by TNFα stimulated microvascular endothelial cells (Manes and Pober, 2008). A murine model has provided further evidence of the importance of TCR stimulation for T cell migration by demonstrating that insulin-specific CD8+ T cells require antigen presentation by endothelial cells for efficient homing to pancreatic islets (Savinov et al., 2003).

The potential for simultaneous chemokine receptor and T cell receptor activation provides a basis to explain chemokine-regulated transendothelial migration of antigen-specific T cells (Ward and Marelli-Berg, 2009). The recent demonstration of a CXCL12-dependent spatial association of CXCR4 with the TCR has clear relevance for the integration of these signals. Indeed, it was shown that CXCR4 can utilise immunoreceptor tyrosine-based activation motifs (ITAMs) on components of the TCR complex for signal transduction (Kumar et al., 2006). Such physical and functional association may also allow TCR activation to influence chemokine receptor signalling, with consequent enhancement or inhibition of migration. Whilst the results of this study are of great interest, they are of little relevance to the migration of T cells into inflammatory sites as CXCR4 is predominantly involved in homeostatic trafficking of naïve T cells to lymph nodes (Campbell et al., 2003).

The chemokine receptor CXCR3 is upregulated on activated T cells and plays a fundamental role in recruiting effector cells, including Th1 cells during inflammatory processes such as allograft rejection (Romagnani, 2005); indeed, CXCR3^−/−^ mice are resistant to acute cardiac allograft rejection (Hancock et al., 2000). This chemokine receptor is activated by at least three inflammatory chemokines: CXCL9 (monokine induced by IFNγ; MIG), CXCL10 (IFNγ-inducible protein; IP-10), and CXCL11 (IFN-inducible T cell α-chemoattractant; I-TAC). A recent study has suggested that, like activated CXCR4 (Kremer et al., 2003), ligand activation of CXCR3 induces phosphorylation of the 70 kD tyrosine kinase, Zeta-associated protein (ZAP-70) which is also a critical component of TCR signal transduction (Dar and Knechtle, 2007). Importantly, it has been suggested that this provides a mechanism for cross-talk between CXCR3 and the TCR, with activation of the TCR modulating the potential for CXCR3-induced signalling, resulting in reduced chemotaxis within a CXCL10 concentration gradient produced by solute diffusion (Dar and Knechtle, 2007).

This study was designed to investigate further the regulation of CXCL10-induced transendothelial migration of CXCR3-expressing human T cells by defining the roles of endothelial cell activation with proinflammatory cytokines with and without concurrent TCR activation in the absence of B7-mediated costimulation. A series of sensitive flow cytometric fluorescence resonance energy transfer (FRET) experiments was then performed to examine the potential for CXCL10 to induce a spatial association between CXCR3 and the TCR complex.

## 2 Methods

### 2.1 EA.hy926 cells

The model human endothelial cell line EA.hy926 (Edgell et al., 1983) was expanded in 75 cm^2^ tissue culture flasks (Corning, UK) containing Dulbecco's Modified Eagles Medium supplemented with 10% FBS (both from Sigma, Poole, UK).

### 2.2 Activated T cells

Peripheral blood mononuclear cells were obtained by density gradient centrifugation of heparinized blood samples taken from healthy adult volunteers. Ficoll-Hypaque density gradient solution (Amersham Biosciences, UK) was used, with mononuclear cells being recovered from the interfacial layer following centrifugation at 400 g for 25 min. These cells were then incubated horizontally in 75 cm^2^ flasks for 1 h in RPMI1640 medium (Sigma) supplemented with 10% FBS, after which the monocyte depleted peripheral blood lymphocyte (PBL) population was recovered.

The PBL were activated and mitogenically expanded by incubation for 72 h in 24-well plates which had previously been coated with 1 μg/ml of the monoclonal mouse anti-human CD3 antibody OKT3 in PBS (IgG2a; Janssen-Cilag Ltd., UK). ^3^H-thymidine incorporation assays were performed in 96-well plates to assess PBL proliferation in response to OKT3 and CXCL10. Bound and soluble CXCL10 were contrasted for their ability to elicit proliferation by first binding 10 nM chemokine to the plate overnight with variable quantities of OKT3, or by adding 10 nM CXCL10 to the medium with the PBL. Following optimal 72 h incubation, 1 μCi ^3^H-thymidine (Amersham Biosciences) was added to each well for 6 h. Cells were lysed, harvested onto a fibre glass filter mat, and β-scintillation counting was performed (Microbeta; PerkinElmer, USA). The data were analysed using Prism 3 software (Graphpad Software, USA).

### 2.3 CFSE staining

For some experiments, activated T cells were labelled with the fluorescent intracellular dye carboxyfluorescein diacetate succinimidyl ester (CFSE). T cells were suspended in serum free RPMI1640 medium containing an optimal concentration of 2.5 μM CFSE (Invitrogen, UK) with agitation for 10 min at room temperature. The cells were then washed twice and resuspended in RPMI complete medium, with satisfactory staining being confirmed by flow cytometry before the cells were used.

### 2.4 CD3 crosslinking prior to chemotaxis

To model the effects of cognate antigen recognition on the endothelium, a system of CD3 crosslinking was used. Cells were harvested 72 h after initial mitogenic expansion by CD3 crosslinking by plate-bound OKT3, washed and resuspended in medium containing OKT3 at a concentration of 1 μg/ml for 20 min. The cells were then washed and divided into two groups. The first was labelled with CFSE as described above. The second was subjected to crosslinking of CD3 by the addition of an Alexa 633-conjugated goat anti-mouse IgG antibody (Invitrogen, UK). This secondary crosslinking process was performed at a variety of different time points before cells were washed and used in the chemotaxis assays described below.

### 2.5 Phenotypic analysis by flow cytometry

Expression of the chemokine receptor CXCR3 by activated T cells was assessed by flow cytometric analysis following staining with a FITC-conjugated mouse anti-human CXCR3 antibody (R&D Systems, Abingdon, UK). The results were compared with those of unstained cells and those of cells stained with a FITC-conjugated IgG1 isotype control antibody (R&D Systems).

### 2.6 Chemotaxis assays and flow cytometric analysis of migrant T cell populations

The migratory potential of activated T cells was assessed using an *in vitro* model of transendothelial migration. EA.hy926 cells were grown to confluence on 5 μm pore polycarbonate membranes (Transwell inserts; Corning Costar, UK) in 24-well plates. For some experiments confluent monolayers were pre-treated with medium containing 100 ng/ml TNFα and 100 ng/ml IFNγ (both from R&D Systems) for 24 h prior to the assay. A volume of 600 μl of RPMI1640 complete medium containing CXCL10 at a concentration of 1 or 10 nM was added to the lower chamber of each well. A total of 1 × 10^6^ activated T cells was then added above the endothelial monolayer. To assess the effect of reactivation of CD3, approximately equal numbers of CFSE labelled T cells (not CD3 crosslinked) and Alexa 633 labelled (CD3 crosslinked) T cells were added to the upper chamber of the well. After incubation for 90 min at 37 °C, migrant T cells were harvested from the lower chamber.

Flow cytometric analysis allowed differentiation between the CFSE labelled and Alexa 633 labelled T cell populations in cell samples added to the upper chamber and retrieved from the lower chamber of the Transwell culture system. These cells were quantified by reference to a known number of fluorescent spheres (Flow Count; Beckman Coulter, UK) which were added to each sample. The ratio of CFSE to Alexa 633 labelled cells was then calculated and normalised relative to a 1:1 value for the pre-migration cell population. Examination of the value for cells recovered from the lower chamber allowed the effect of CD3 crosslinking on the migratory ability of CXCL10 responsive T cells to be determined.

Additional experiments were performed in which the polycarbonate membranes were removed from the culture inserts at various time points after addition of the T cells and fixed for examination by scanning laser confocal microscopy followed by X–Z analysis of the image stack.

### 2.7 Fluorescence resonance energy transfer (FRET)

To investigate any possible interaction between the chemokine receptor CXCR3 and the TCR complex, a flow cytometric FRET assay was performed (Kumar et al., 2006). Activated T cells were prepared as described above, some were incubated with 10 nM CXCL10 for 20 min at 37 °C and then the cells were stained with fluorochrome-conjugated antibodies specific for CXCR3 (clone 49801, PE conjugate; R&D Systems) and CD3ɛ (clone UCHT1, APC conjugate; Becton Dickinson). The potential for FRET was assessed by flow cytometry with excitation of CXCR3-PE at 488 nm and measurement of emission from CD3ɛ-APC at 675 nm. To demonstrate that the FRET was a non-random event both antibodies were titrated to a saturating concentration as recommended by Batard et al. (2002). Control experiments were performed in which either the incubation with CXCL10 was omitted, or anti-CD3 was substituted with APC conjugated anti-CD45 antibodies as described previously (Kumar et al., 2006). To determine whether any FRET detected was occurring intracellularly following endocytosis of receptor complexes, a 5 min acid wash (20 mM HCl/HBSS; pH 2) at 4 °C was performed after CXCL10 stimulation to remove surface antibodies. Identical assays were performed using conjugated antibodies specific for CCR2 (clone 48607, PE conjugate; R&D Systems) and CD3ɛ, and the specific chemokine ligand CCL7. In all cases FRET was examined and energy transfer efficiencies calculated as described by Batard et al. (2002).

## 3 Results

### 3.1 Analysis of CXCR3 expression by activated T cells

**Fig. 1:**
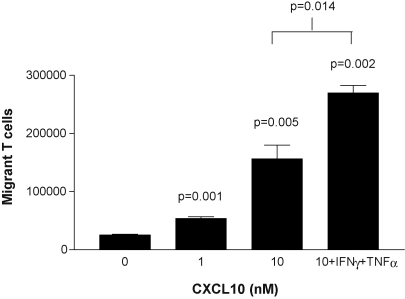
Analysis of the function of CXCR3. Chemotactic migration of activated T cells through an EA.hy926 endothelial cell monolayer towards various concentrations of CXCL10. Prior activation of the endothelium with IFNγ and TNFα increased the number of migrant cells. Representative results are shown from one of three similar assays; the bars show mean values ± SEM.

Fig. 1 demonstrates that T cells activated for 72 h by mitogenic expansion by CD3 crosslinking are highly responsive to stimulation of the chemokine receptor CXCR3; this receptor was not expressed on freshly isolated T cells (not shown). Chemotaxis assays were performed in the presence of CXCL10. Fig. 1 shows results from experiments to optimise conditions for these assays. CXCL10 was chemotactic for activated T cells, with subendothelial concentrations as low as 1 nM causing a statistically significant increase in migration (*p* < 0.001). A greater migratory response was produced by 10 nM CXCL10 but this was increased further by prior activation of the endothelial monolayer with IFNγ and TNFα (*p* = 0.014).

### 3.2 CXCR3 and CD3 synergistically activate T cells

**Fig. 2:**
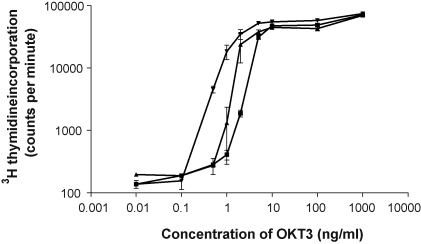
CXCR3 and CD3 activation have synergistic effects on T cell proliferation. A range of OKT3 concentrations were bound to a 96-well plate overnight. 10 nM CXCL10 was also bound to the plate overnight (-▾-), added in solution to the lymphocyte suspension (-▴-), or controlled with PBS (-■-). The assay was incubated for 72 h and T cell proliferation was determined by measuring ^3^H-thymidine incorporation. Representative results are shown from one of three similar assays; the bars show mean values ± SEM.

Crosslinking CD3 with immobilised OKT3 induces T cell activation and proliferation. A titration of OKT3 was performed with and without the addition of CXCL10 to determine whether CXCR3 ligation alters the proliferative response after incubation for 72 h. Fig. 2 shows that addition of CXCL10 to the culture medium (soluble CXCL10) resulted in a moderate increase in T cell proliferation in the presence of a suboptimal concentration of OKT3; this was increased further by binding the chemokine to the plate. Non-linear regression demonstrated that half-maximal T cell proliferation was produced by 5 ng/ml OKT3 in the absence of chemokine, but this was reduced to 1.9 ng/ml (*p* < 0.05) in the presence of bound CXCL10. The chemokine was not mitogenic in the absence of CD3 stimulation (data not shown).

### 3.3 Evaluation of a transendothelial chemotaxis assay using activated T cells

**Fig. 3:**
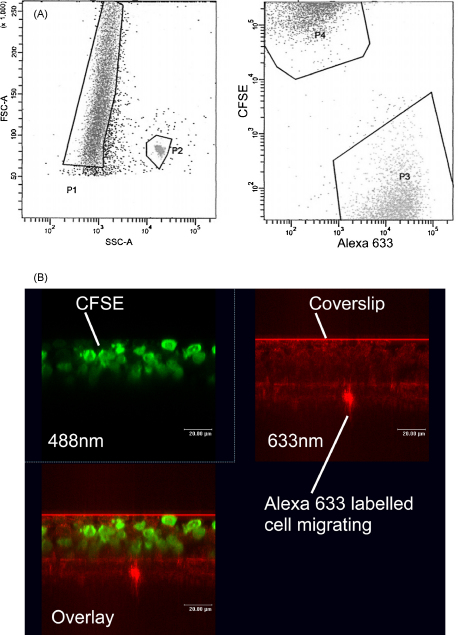
Validation of the transendothelial T cell migration model. (A) Representative flow cytometric dotplots showing analysis of transendothelial chemotaxis assays. Region P1 contains the total T cell population retrieved from the bottom chamber of a well following transendothelial migration. P2 contains the fluorosphere counting beads added to the sample. P3 contains the Alexa 633 labelled T cells within P1. P4 contains the CFSE labelled T cells within P1. Enumeration of events in these regions allowed the number of CFSE and Alexa 633 labelled cells to be calculated for analysis as described in Section 2. (B) Confocal microscopy showing the migration of T cells through cytokine-stimulated endothelium. By separate use of lasers at 488 nm and 633 nm wavelengths it was possible to visualise CFSE labelled (untreated) T cells (green) and Alexa 633 labelled (CD3 crosslinked) T cells (red) in separate images. Overlaying these images allowed the spatial relationship of both cell populations to be compared at different time points. The representative image was captured after 120 min and shows an Alexa 633 labelled cell in the process of migration through the membrane, whilst CFSE labelled cells remain on the surface of the endothelial cell monolayer. (For interpretation of the references to color in this figure legend, the reader is referred to the web version of the article.)

Having found CXCL10 to be chemotactic for activated T cells, experiments were performed to determine whether labelling with the intracellular dye CFSE would adversely affect the migration of activated T cells. No significant difference (*p* = 0.9) was observed between transendothelial migration of unstained and CFSE-stained T cells towards 10 nM CXCL10 (data not shown). Fig. 3A shows how flow cytometric analysis of the migrant cell population was performed. The migrant cells (left panel, P1) were mixed with a known number of beads (left panel, P2) for counting. The cells were also separated into CFSE positive cells (P4) and Alexa 633 positive cells (P3) in order to assess the effect of crosslinking CD3 immediately prior to performing the chemotaxis assay.

Scanning laser confocal microscopy was used to examine migration of activated T cells through a cytokine-stimulated endothelium. This technique allowed direct visualisation of migrating cells by X–Z cross-sectional analysis of the endothelial cell monolayer and the Transwell membrane. Equal numbers of CFSE labelled T cells and Alexa 633 labelled cells which had been reactivated by CD3 crosslinking for 90 min were applied at the start of the chemotaxis assay. Fig. 3B shows the distribution of CFSE labelled cells (green) and Alexa 633 labelled cells (red) in a filter sampled 120 min after addition of the T cells. An Alexa 633 labelled cell can be seen passing through the polycarbonate membrane, whilst large numbers of CFSE labelled cells remain on the apical surface of the endothelium.

### 3.4 Transendothelial migration of activated T cells through endothelial monolayers

**Fig. 4:**
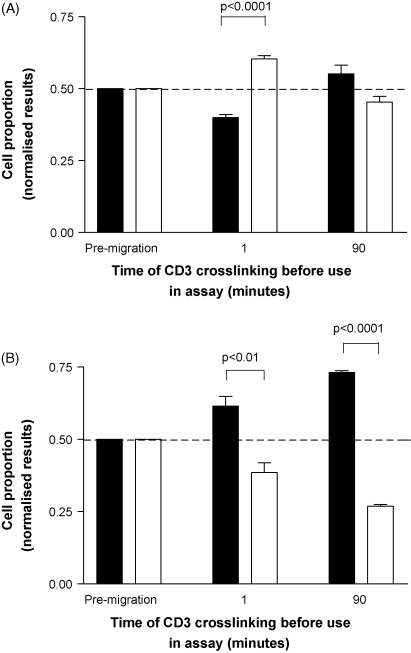
The effect of CD3 crosslinking on chemotaxis of activated T cells through an endothelial monolayer. In both graphs, the bars indicate the proportion of Alexa 633 (crosslinked; filled bar) and CFSE labelled (untreated; open bar) T cells. (A) Using resting endothelial cells. A significant reduction in the proportion of Alexa 633 labelled cells was seen in the migrant population, when CD3 crosslinking was performed 1 min before the assay. When CD3 crosslinking was carried out 90 min before the assay there was no significant change in the proportion of these cells in the migrant population. (B) Using endothelial cells which had been previously stimulated with IFNγ and TNFα. Significant increases in the proportion of Alexa 633 labelled cells are seen in the migrant cell populations following CD3 crosslinking for either 1 min or 90 min. In both cases representative results are shown from one of three similar experiments; the bars show mean values ± SEM.

Fig. 4 shows the results of experiments to determine the effect of CD3 crosslinking on transendothelial migration of activated T cells through endothelial monolayers towards CXCL10. Fig. 4A shows results for resting endothelial cells. It can be seen that crosslinking CD3 for 1 min before the assay caused a significant reduction in the migration of Alexa 633 labelled cells when compared to the migration of the untreated, CFSE labelled cells. Interestingly, this retardation of migration was only transient, with no significant difference seen between the two T cell populations when CD3 crosslinking was performed 90 min before the assay.

Fig. 4B shows the results of experiments using endothelial monolayers which had been previously stimulated with IFNγ and TNFα. It can be seen that crosslinking CD3 for 1 min before the assay caused a statistically significant increase in the migration of the Alexa 633 labelled cells when compared to the migration of the untreated, CFSE labelled cells (*p* < 0.01). This differential migration became even more pronounced when CD3 crosslinking was performed 90 min before the assay (*p* < 0.0001); after this time the untreated T cells constituted only 25% of the migrant population.

### 3.5 Demonstration of an association between CXCR3 and CD3 by measuring FRET

**Fig. 5:**
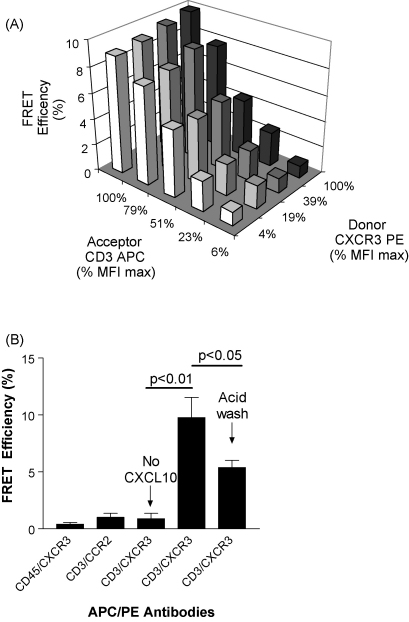
CXCR3 can be spatially associated with CD3. (A) Mean FRET efficiencies were calculated for titrations of anti-CXCR3-PE and anti-CD3-APC demonstrating that the FRET seen is non-random. Fluorescence intensity is expressed as a percentage of the maximal median fluorescence intensity (MFI). The increasing percentage values corresponded to increasing anti-CD3ɛ antibody concentrations. (B) Control experiments demonstrated that the FRET signal is not due to non-specific spectral overlap (CD45/CXCR3 control), is specific for CXCR3 (CCR2/CD3 control), is ligand-dependent and is not entirely a consequence of receptor internalisation (reduction following acid wash). The bars show mean values ± SEM.

Fig. 5 shows representative results from experiments using FRET to demonstrate an association between CXCR3 and CD3ɛ using antibodies conjugated respectively with PE and APC (Batard et al., 2002; Kumar et al., 2006). A positive FRET signal using this combination of fluorophores is indicated when the efficiency of energy transfer is greater than 1% (Batard et al., 2002). Fig. 5A shows that for a given titration of the acceptor antibody (anti-CD3-APC) the median fluorescence intensity (MFI) of the FRET signal was insensitive to increasing the concentration of donor antibody (anti-CXCR3-PE).

As sensitisation of the acceptor was measured, FRET efficiency should be independent of donor concentration for a particular acceptor concentration. This is because FRET efficiency is calculated relative to the donor according as described by Batard et al. (2002). The absence of an increase in FRET efficiency as donor concentration increases is indicative of a positive FRET signal and indicates a close (<10 nm) association between CXCR3 and CD3. Importantly, an acid wash reduced significantly this FRET signal (Fig. 5B), demonstrating that this spatial association occurs on the T cell-surface.

In control experiments (Fig. 5B) no FRET was observed in the absence of CXCL10 treatment, or when the cells were labelled with appropriately conjugated antibodies specific for CXCR3 and CD45. Further experiments also demonstrated the absence of FRET between immunofluorescently labelled CD3ɛ and CCR2 following specific T cell stimulation with the specific ligand 10 nM CCL7 (Fig. 5B).

## 4 Discussion

This study was performed to assess downstream functions of the potential synergy between signalling through CXCR3 and the TCR on activated T cells. Initial studies demonstrated that resting human T cells expressed little CXCR3, but this receptor was expressed by the majority of T cells after mitogenic activation for 72 h by culturing with the immobilised CD3 antibody, OKT3. Indeed, previous studies have shown that CXCR3 is stored by resting T cells in cytoplasmic granules allowing release onto the cell-surface within minutes of activation (Gasser et al., 2006). This chemokine receptor was shown to function in transendothelial chemotaxis assays in which the ligand CXCL10 was added below the endothelial cell monolayer. In these experiments it was found that activation of the endothelial monolayer with the proinflammatory cytokines IFNγ and TNFα greatly increased the number of migrant cells. These data are consistent with previous studies showing increased CXCR3 expression during T cell activation, with this receptor being most highly expressed by memory and effector Th1 cells (Loetscher et al., 1996).

The transendothelial migration induced by subendothelial addition of CXCL10 suggests recognition of this ligand on the apical surface of the endothelial cells, where it may be bound and presented by heparan sulphate (Ranjbaran et al., 2006). The marked increase in T cell migration observed across activated endothelium is likely to be due to increased expression of a range of factors (Sana et al., 2005). These include increased endothelial production of additional chemokines such as CCL2 (Hardy et al., 2004), the increased potential of modified heparan sulphate to bind and present chemokines on the apical surface of the activated endothelial cells (Carter et al., 2003), and increased expression of the critical T cell adhesion molecules ICAM-1 and VCAM-1 (Alon and Ley, 2008).

The induction of T cell proliferation by OKT3 antibodies has been well described and is a specific feature of this IgG2a monoclonal CD3 antibody (Van Wauwe et al., 1980); the mitogenic activity is produced by crosslinking the receptor complex. The current study demonstrated that 50% maximal T cell proliferation was produced by treatment of the culture wells with an antibody concentration of approximately 5 ng/ml, which is similar to the mitogenic concentration defined in earlier studies (Van Wauwe et al., 1980). However, prior immobilisation of CXCL10 onto the culture wells induced a similar rate of T cell proliferation in the presence of OKT3 at only 1.9 ng/ml. This observation of CXCL10-mediated costimulation is consistent with a previous study which demonstrated that CXCR3 deficient T cells respond poorly in allogeneic mixed leukocyte culture (Hancock et al., 2000).

It has been reported that stimulation with CXCL12 (Nanki and Lipsky, 2000) or CCL5 (Bacon et al., 1995) will both enhance T cell proliferation and cytokine production. Although the mechanism for this chemokine-mediated costimulation remains incompletely defined, it has been shown that both CXCR4 and CCR5 migrate to the immune synapse following activation (Contento et al., 2008); this is associated with reduced T cell migration allowing increased contact with antigen presenting cells (Molon et al., 2005). It is also known that stimulation with CXCL12 induces a spatial association between CXCR4 and the TCR complex allowing the chemokine receptor to activate components of the TCR signalling pathway leading to increased CD69 and IL-2 production (Kumar et al., 2006). A similar utilisation of the TCR signalling pathway has been shown for CXCR3 (Dar and Knechtle, 2007).

An important validation experiment demonstrated that T cells labelled with CFSE showed a normal chemotactic response towards CXCL10. This result is consistent with previous reports indicating that limited concentrations of CFSE do not impair cell motility (De Clerck et al., 1994). A sensitive flow cytometric assay was developed to allow simultaneous enumeration of T cells which had been either activated with plate-bound OKT3 for 72 h or activated for 72 h and then subjected to a further cycle of CD3 crosslinking to model B7-deficient antigen presentation by endothelial cells (Al-Lamki et al., 2008; Manes et al., 2007). Application of these techniques demonstrated that CD3 activation for 1 min before measuring chemotaxis towards CXCL10 across a resting endothelial monolayer reduced the migration of these cells in comparison with T cells which had not been activated at the time of the assay. However, this retardation was transient as T cells which had been activated for 90 min before the assay showed an equal migration to cells which had not been activated. This observation is consistent with previous reports that specific TCR activation can arrest T cell migration across non-IFNγ treated endothelial cell monolayers by activation of Src family tyrosine kinases (Manes et al., 2007).

Endothelial cell monolayers which have been pre-treated with the proinflammatory cytokines IFNγ and TNFα support greater chemotactic responses. However, T cells which had been activated by crosslinking CD3 for either 1 min or 90 min showed a further increase in migration towards CXCL10 across activated endothelium when compared with cells which had not been reactivated. Indeed, T cells which had been reactivated for 90 min before the assay constituted almost 75% of the migrant population. Although the basis for the difference in migration of reactivated T cells across resting and activated endothelial cells is unclear, a previous report has noted that stimulation of endothelial cells with IFNγ can increase the migration of antigen-activated T cells by reversing a “stop signal” (Tay et al., 2003). Studies performed *in vivo* have also shown that IFNγ is required for the homing of antigen-specific T cells during autoimmune diabetes (Savinov et al., 2001), with endothelial presentation of autoantigen promoting the recruitment of specific T cells (Greening et al., 2003).

A series of experiments was performed to identify any change in the spatial relationship between CXCR3 and CD3 following chemokine treatment in order to identify a basis for the synergy observed between these receptors. An established flow cytometric FRET assay was employed for this study using the fluorochromes PE and APC, which transfer fluorescence energy when they are separated by a distance of less than 10 nm (Batard et al., 2002). Treatment of activated T cells with 10 nM CXCL10 markedly increased the fluorescence output due to FRET when CXCR3 and CD3ɛ were labelled with antibodies conjugated to these fluorochromes. These data indicate for the first time a ligand-dependent, spatial association between CXCR3 and the TCR complex.

A similar increase in FRET was not observed when the PE and APC fluorochromes were bound to CXCR3 and the common cell-surface molecule CD45, indicating that the interaction between CXCR3 and CD3ɛ was induced specifically by CXCL10. Importantly, a brief acid wash to remove cell-surface antibodies diminished the FRET signal demonstrating that these receptors were spatially associated on the cell-surface. The remaining, acid resistant FRET signal is indicative of a potentially non-specific fluorochrome association within the endosomal compartment following internalisation. These FRET results are consistent with a previous demonstration that CXCL12 induces a physical association between CXCR4 and CD3 on the surface of T cells (Kumar et al., 2006). However, ligand-induced chemokine receptor association with the TCR complex is not a general finding as treatment of the activated T cells with CCL7 did not induce FRET between CCR2 and CD3ɛ.

This study has shown that stimulation of CXCR3 on the surface of activated T cells produces signals which can synergise with those transduced by activation of the TCR complex. These signals enhance both T cell proliferation and the specific migration of T cells across cytokine-activated endothelium. The demonstration of a chemokine ligand-induced spatial association between CXCR3 and CD3ɛ is consistent with a previous demonstration that the TCR and CXCR3 chemokine receptor signalling pathways share signal transduction molecules (Dar and Knechtle, 2007) and suggests a mechanistic basis for the enhanced transendothelial migration observed in this study.

## Conflict of interest

The authors have no financial or commercial conflicts of interest to disclose.
